# CYP1B1 Enhances Cell Proliferation and Metastasis through Induction of EMT and Activation of Wnt/β-Catenin Signaling via Sp1 Upregulation

**DOI:** 10.1371/journal.pone.0151598

**Published:** 2016-03-16

**Authors:** Yeo-Jung Kwon, Hyoung-Seok Baek, Dong-Jin Ye, Sangyun Shin, Donghak Kim, Young-Jin Chun

**Affiliations:** 1 College of Pharmacy, Chung-Ang University, Seoul, Korea; 2 Department of Biological Sciences, Konkuk University, Seoul, Korea; National Cancer Center, JAPAN

## Abstract

Cytochrome P450 1B1 (CYP1B1) is a major E_2_ hydroxylase involved in the metabolism of potential carcinogens. CYP1B1 expression has been reported to be higher in tumors compared to normal tissues, especially in hormone-related cancers including breast, ovary, and prostate tumors. To explore the role of CYP1B1 in cancer progression, we investigated the action of CYP1B1 in cells with increased CYP1B1 via the inducer 7,12-dimethylbenz[α]anthracene (DMBA) or an overexpression vector, in addition to decreased CYP1B1 via the inhibitor tetramethoxystilbene (TMS) or siRNA knockdown. We observed that CYP1B1 promoted cell proliferation, migration, and invasion in MCF-7 and MCF-10A cells. To understand its molecular mechanism, we measured key oncogenic proteins including β-catenin, c-Myc, ZEB2, and matrix metalloproteinases following CYP1B1 modulation. CYP1B1 induced epithelial-mesenchymal transition (EMT) and activated Wnt/β-catenin signaling via upregulation of *CTNNB1*, *ZEB2*, *SNAI1*, and *TWIST1*. Sp1, a transcription factor involved in cell growth and metastasis, was positively regulated by CYP1B1, and suppression of Sp1 expression by siRNA or DNA binding activity using mithramycin A blocked oncogenic transformation by CYP1B1. Therefore, we suggest that Sp1 acts as a key mediator for CYP1B1 action. Treatment with 4-hydroxyestradiol (4-OHE_2_), a major metabolite generated by CYP1B1, showed similar effects as CYP1B1 overexpression, indicating that CYP1B1 activity mediated various oncogenic events in cells. In conclusion, our data suggests that CYP1B1 promotes cell proliferation and metastasis by inducing EMT and Wnt/β-catenin signaling via Sp1 induction.

## Introduction

Cytochrome P450 1B1 (CYP1B1) belongs to the CYP1 family and shares enzymatic activities with two other CYP1 family members, CYP1A1 and CYP1A2 [1]. It primarily acts as a hydroxylase for 17β-estradiol at positions C2 and C4, and the products from these enzymatic reactions participate in metabolic processes that generate quinone metabolites involved in the production of carcinogenic DNA adducts [2–4].

CYP1B1 is normally expressed in most tissues. However, its expression is elevated in tumors compared to normal tissues [5–8], especially in hormone-related cancers including breast, ovary, and prostate tumors [9–11] and the pre-disposing potential of CYP1B1 for various cancers also has been widely reported [12–14]. Recently, it has been suggested that CYP1B1 enhances cell proliferation by inducing cell cycle transition and inhibiting cellular apoptosis in endometrial and breast cancer cells [15, 16]. Moreover, CYP1B1 polymorphisms have been implicated as risk factors in various cancers, and CYP1B1-mediated carcinogenesis may depend on CYP1B1 enzymatic activity [17–19]. Taken together, these findings suggest that CYP1B1 might be a driver in cancer progression and, therefore, represent a significant cancer biomarker and potential target for anticancer therapy. However, a detailed molecular mechanism describing CYP1B1-mediated oncogenesis remains unknown.

β-catenin plays an important role as a key mediator in the Wnt/β-catenin signaling pathway. Following activation by Wnt ligand-receptor binding, β-catenin escapes proteosomal degradation and translocates into the nucleus, where it binds its target genes and promotes multiple pathways involved in carcinogenesis [20, 21].

Several studies have suggested that Wnt/β-catenin signaling may be related to epithelial-mesenchymal transition (EMT) because they both require β-catenin. In normal tissues, cells establish tight junctions using cell membrane glycoproteins like E-cadherin [22, 23]. Adjacent epithelial-like cells bind to one another via cell-surface E-cadherins, which are linked to the actin cytoskeleton or cytoplasmic cell signaling components including α-, γ-, and β-catenin [24]. During carcinogenesis, E-cadherin repressors including SNAIL, ZEB1/2, and TWIST are upregulated, which causes the loss of E-cadherin and subsequent induction of EMT [25, 26]. Following E-cadherin suppression, β-catenin is released from E-cadherin-catenin-actin complexes and accumulates in the cytosol and nucleus, which allows it to act independently or synergistically with the Wnt/β-catenin signaling pathway [27–29]. Therefore, EMT and Wnt/β-catenin signaling may act synergistically during carcinogenesis.

In the present study, we explored the role of CYP1B1 in carcinogenesis and cancer progression including the molecular mechanism that drives CYP1B1-mediated oncogenesis. To do so, we measured multiple hallmarks of cancer progression including cell proliferation, invasion, and migration following CYP1B1 induction or inhibition. We further investigated the key factors driving cell proliferation and invasion following CYP1B1 modulation and found several target proteins that are related to EMT and Wnt/β-catenin signaling. To the best of our knowledge, these findings establish the molecular mechanisms driving CYP1B1-mediated oncogenesis for the first time.

## Materials and Methods

### Reagents

7,12-Dimethylbenz[*α*]anthracene (DMBA), mitomycin C, mithramycin A, 4-hydroxyestradiol, 2-hydroxyestradiol, and charcoal-stripped FBS were purchased from Sigma (St. Louis, MO, USA). 2,2′,4,6′-Tetramethoxystilbene (TMS) was kindly provided by Dr. Sanghee Kim (Seoul National University, Seoul, Korea). Rabbit polyclonal antibody for E-cadherin was purchased from Millipore (Bedford, MA, USA). M-MLV reverse transcriptase and RNase inhibitor were purchased from Promega (Madison, WI, USA). Ex Taq Polymerase was obtained from TaKaRa Bio (Shiga, Japan). SYBR green was purchased from QIAGEN (Hilden, Germany). Rabbit polyclonal antibodies for CYP1B1, Sp1, β-catenin, E-cadherin, cyclin D1, vimentin, SNAI1, and GAPDH; mouse monoclonal antibody for ZEB2 and c-Myc; Texas Red-conjugated goat anti-rabbit IgG; and UltraCruz^TM^ Mounting Medium were purchased from Santa Cruz Biotechnology (Santa Cruz, CA, USA). HRP-conjugated goat anti-rabbit IgG and DyLight^®^ 594-conjugated goat anti-mouse were obtained from Bethyl (Montgomery, TX, USA) and mouse monoclonal antibody for PCNA was purchased from Cell Signaling Technology (Beverly, MA, USA). Other chemicals and reagents were of the highest quality commercially available.

### Cell culture

MCF-7, MDA-MB-231, and HeLa cells were obtained from the Korean Society Cell Bank (KCLB), and MCF-10A cells were kindly provided by Dr. Aree Moon (Duksung Women’s University, Seoul, Korea). Authentication of cells has been performed by KCLB based on DNA fingerprinting analysis using short tandem repeat analysis. MCF-7 and MDA-MB-231 cells were cultured in RPMI medium supplemented with 10% (v/v) heat-inactivated FBS, 100 U/ml penicillin, and 100 μg/ml streptomycin. HeLa cells were cultured in MEM medium supplemented with 10% (v/v) heat-inactivated FBS, 100 U/ml penicillin, and 100 μg/ml streptomycin. MCF-10A cells were cultured in monolayer as described previously [30]. For treatment of MCF-7 cells with 4-OHE_2_ or 2-OHE_2,_ 1×10^6^ cells were seeded in growth media as a monolayer onto 100-mm dish plates and maintained at 37°C in a humidified atmosphere with 5% CO_2._ After 24 h, the media was changed to phenol red-free RPMI (Thermo Scientific, IL, USA) with 10% (v/v) charcoal-stripped FBS, 100 U/ml penicillin, and 100 μg/ml streptomycin. Cells were maintained for 72 h and were subsequently provided fresh media containing designated concentrations of 4-OHE_2_ or 2-OHE_2_. After 48 h, cells were harvested and processed for further studies.

### Transient transfection of plasmid DNA and siRNA

CYP1B1-specific siRNA (target sequence: CAGCATGATGCGCAACTTCTT, Qiagen) and the overexpression vector pcDNA 3.1/Zeo containing the CYP1B1-encoding sequence were used in transfections. Cells were transfected at room temperature with 37.5 nM siRNA or 8 μg plasmid with the Neon Transfection System (Invitrogen, Carlsbad, CA, USA) and cultured in 100-mm dishes in antibiotic-free RPMI with 10% FBS for 48 h.

### Adenovirus infection

The infection of adenovirus carrying CYP1B1-ORF genes (ViGene Biosciences Inc., Rockville, MD, USA) was performed in serum-free media at an m.o.i. of 750 vp (virus particles)/cell for MCF-7 cells. After 24 h, media change was carried out with serum-containing fresh media. Cells were maintained at 37°C in a humidified atmosphere with 5% CO_2_ for 24 h and harvested or fixed for further studies. Under these circumstances, the transduction efficiency of the CYP1B1 gene carrying adenovirus reached almost 100%.

### Cell viability assay

CYP1B1-overexpressed cells (1×10^4^ cells/well) were plated onto 96-well plates and incubated in 37°C. After stabilization for 48 h, 10 μl EZ-CyTox (Daeil Lab Service, Seoul, Korea) was added to each well and incubated for 2 h at 37°C. Formazan formation was quantified by spectrophotometry at 450 nm using a Sunrise^™^ microplate reader (Tecan, Männedorf, Switzerland). Each experiment was performed at least three times independently.

### Subcellular fractionation

Subcellular fractionation was performed using the NE-PER^®^ Nuclear and Cytoplasmic Extraction kit from Thermo Scientific. Western blot analyses were carried out using antibodies against the following control marker proteins: β-actin for the cytosolic fraction and Hsp70 for the nuclear fraction.

### Invasion assay

Cell invasion was measured using the QCM^™^ 24-well Cell Invasion Assay Kit (Millipore), according to the manufacturer’s instructions. Briefly, cells were seeded onto insert chambers containing a collagen-coated polycarbonate membrane with 8-μm pores. Cells that invaded the ECM layer were stained with 4′,6-diamidino-2-phenylindole (DAPI). Invading cells in five fields per chamber were visualized and counted under the LSM700 Confocal Laser Scanning Microscope (Carl Zeiss, Jena, Germany). Each experiment was performed three times independently.

### Wound healing assay

Cells (1×10^6^ cells/well) were cultured in 6-well culture plates. After 24 h, cells with 90% confluence were washed with PBS and treated with mitomycin C (25 μg/ml) for 30 min. After washing, a single wound per monolayer was created using sterile pipette tips. Plates were photographed after the indicated time. Each experiment was performed at least three times independently.

### Quantitative PCR (qPCR)

Total RNA was extracted using Ribospin^™^ (GeneALL, Seoul, Korea). Total RNA (500 ng) was reverse transcribed at 37°C for 1 h in 20 μl total volume containing 5× RT buffer, 10 mM dNTPs, 40 U RNase inhibitor, 200 U Moloney murine leukemia virus reverse transcriptase, and 100 pmol oligo-dT primer. Quantitative PCR (qPCR) was performed using the Rotor-Gene SYBR^®^ PCR Kit, as recommended by the manufacturer, and analyzed using QIAGEN Rotor-Gene Q Series software. Each reaction contained 12.5 μl 2× SYBR^®^ Green PCR Master Mix, 1 μM oligonucleotide primers, and 2 μl cDNA in a final volume of 25 μl. Amplification was conducted as follows: one cycle at 95°C for 5 min, followed by 40 cycles of denaturation at 95°C for 5 seconds and annealing/extension at 60°C for 10 seconds. Primer sequences are listed in S1 Table.

### Western blot

Whole cells were harvested by scraping and lysed in 50 mM Tris-HCl (pH 8.0) containing 150 mM NaCl, 1% nonidet P-40, 1 mM PMSF, 1 μg/ml aprotinin, and 1 μg/ml leupeptin for 30 min followed by centrifugation at 22000×*g* for 15 min at 4°C. Protein concentrations were measured using BCA Protein Assay Reagents (Thermo). Extracted proteins (20 μg) were separated by SDS-PAGE on 10%–12% polyacrylamide gels and electrophoretically transferred onto PVDF membranes. Membranes were blocked with 5% nonfat milk in Tris-buffered saline containing 0.1% Tween-20 for 1 h at 4°C, and then incubated overnight with specific antibodies. After incubating with secondary antibodies for 2 h, proteins were visualized using enhanced chemiluminescence reagents (Thermo). Quantitative data were obtained using Quantity One software (Bio-Rad, Hercules, CA, USA).

### Dual luciferase reporter assay

Cells (2×10^4^ cells/well) were co-transfected with 200 ng of pcDNA 3.1/Zeo CYP1B1, CYP1B1 L432V, CYP1B1 N203S overexpression plasmid and TOP/FOP, ZEB1, TWIST1 or E-cadherin reporter plasmids, according to the manufacturer’s protocol, using Neon^TM^ transfection system (Invitrogen). pRL-renilla (Promega) was co-transfected as control. After 24 h, cells were lysed using passive lysis buffer and luciferase activities were measured with FilterMax F3 (Molecular Devices, LLC, USA) using the Dual Luciferase Assay System (Promega).

### Immunofluorescence

Cells grown on coverslips were treated with the indicated reagent concentrations, rapidly washed with PBS, and fixed with 3.7% (w/v) paraformaldehyde for 30 min at room temperature. After washing with PBS, the cells were blocked for 30 min in PBS containing 5% goat serum and 0.2% Triton X-100, and then incubated with specific primary antibodies overnight. Next, the cells were washed extensively and stained with Texas Red-conjugated goat anti-rabbit IgG or DyLight^®^ 594-conjugated goat anti-mouse IgG (1:500) for 2 h. After additional washes, the coverslips were mounted onto glass slides using UltraCruz^™^ Mounting Medium containing DAPI. Fluorescence signals were analyzed using an LSM700 Confocal Laser Scanning Microscope (Carl Zeiss).

### 7-Ethoxyresorufin-O-Deethylation (EROD) assay

Cells (5×10^5^) were plated in 2 ml of culture medium and incubated for 48 h. After incubation, the cells were harvested by scrapping in ice-cold 0.1 M potassium phosphate buffer (pH 7.4). Cells were centrifuged at 1000×*g* for 5 min at 4°C and the pellets were resuspended in the same buffer. The cells were sonicated for 30 seconds at 4°C. The reaction mixture contained 0.1 M potassium phosphate buffer (pH 7.4), 2 mg/ml bovine serum albumin, 50 pM rabbit NAPDH-P450 reductase, 2 μM ethoxyresorufin, and cellular sonicates. The reaction mixtures were pre-incubated at 37°C for 3 min and the reaction was initiated by addition of 120 μM NADPH. After 20 min of incubation at 37°C in a shaking water bath, the reaction was terminated by addition of 1 ml of ice-cold methanol. The formation of resorufin was determined fluorometrically with FlexiStation 3 (Molecular Devices), with excitation and emission wavelengths of 544 nm and 590 nm, respectively. Protein concentrations were estimated using the BCA Protein Assay Reagents (Thermo) according to the supplier's recommendations.

### Statistical analysis

Statistical analyses were performed using one-way analysis of variance and Dunnett’s Multiple Comparison *t*-test on Graph-Pad Prism Software (GraphPad Software Inc., San Diego, CA). The difference was considered statistically significant when *p* ≤ 0.05.

## Results

### CYP1B1 induces cell proliferation and metastasis

**Fig 1:**
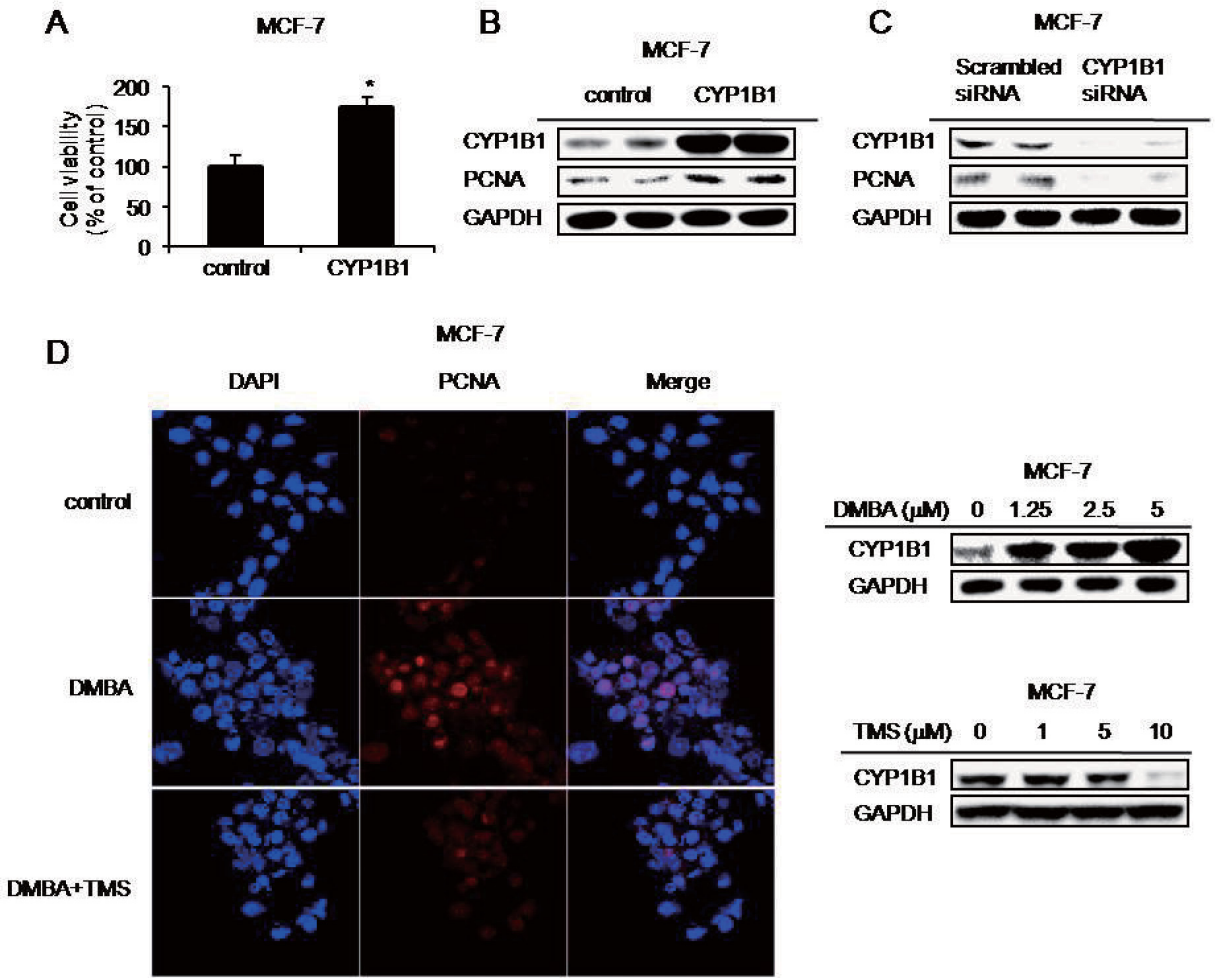
CYP1B1 enhances cell proliferation. (A) Relative cell viability determined by CCK assay subsequent to induction of CYP1B1 in MCF-7 cells following CYP1B1 overexpression. Data are representative of experiments in triplicate. (*p≤0.05) (B-D) PCNA was measured by western blot and confocal microscopy following (B) CYP1B1 overexpression, and (C) CYP1B1 knockdown. (D) Confocal microscopic analysis of MCF-7 cells treated with 5 μM DMBA and 10 μM TMS for 48 h. Cells were pre-treated with TMS for 1 h prior to DMBA.

To explore the role of CYP1B1 in cancer progression, its effects on cell proliferation, migration, and invasion were investigated. CYP1B1 overexpression significantly increased cell proliferation in MCF-7 cells (Fig 1A).

PCNA (Proliferating cell nuclear antigen) has been widely used as a marker for cell proliferation [31]. Accordingly, PCNA protein was upregulated by CYP1B1 overexpression (Fig 1B), while CYP1B1 knockdown had the opposite effect (Fig 1C). Confocal microscopic analysis likewise indicated that DMBA, a CYP1B1 inducer, increased PCNA expression while TMS, a CYP1B1-specific inhibitor, decreased PCNA levels (Fig 1D). These data suggest that CYP1B1 enhances cell proliferation through PCNA expression.

**Fig 2:**
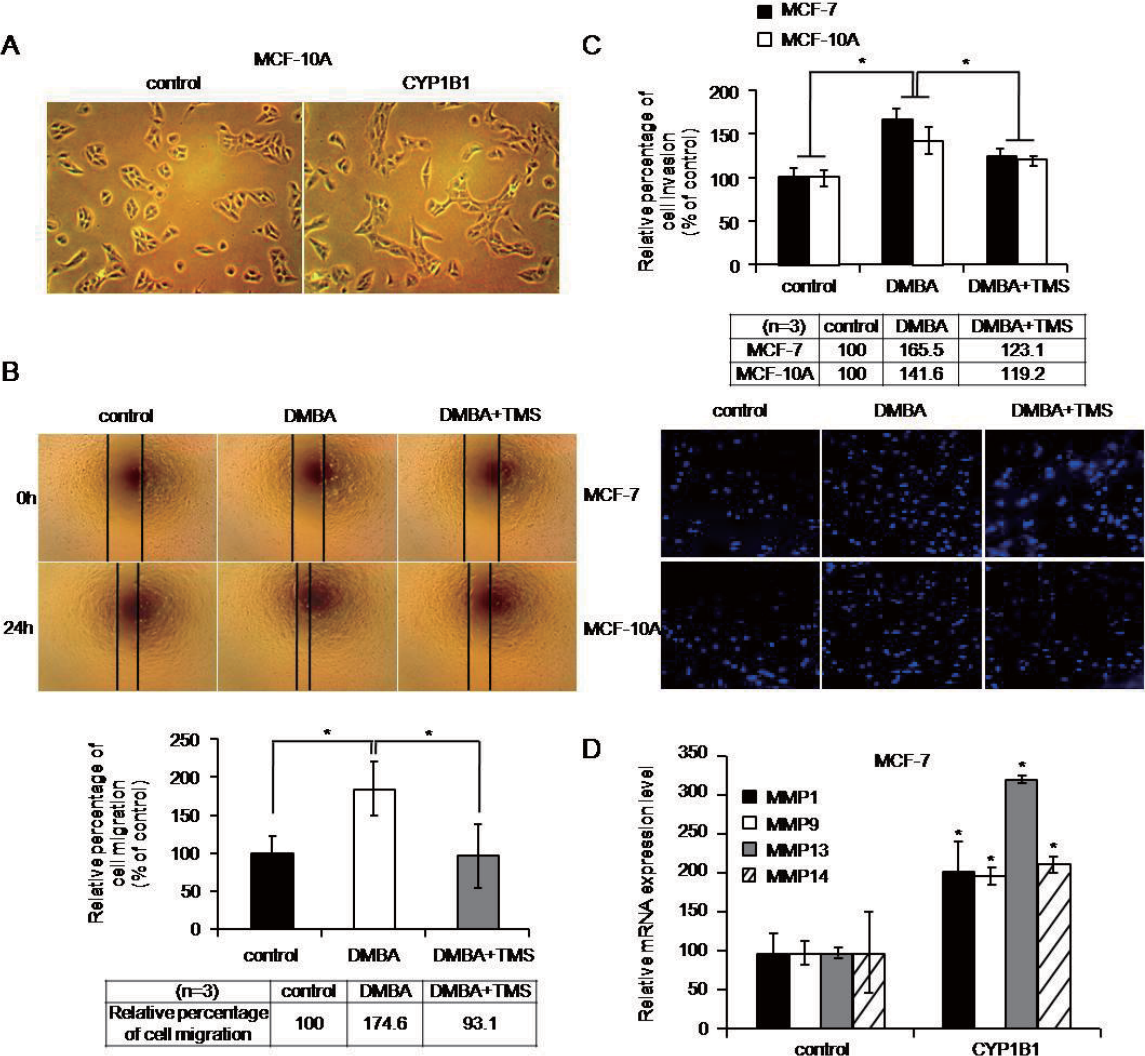
CYP1B1 induces cell migration and invasion. (A) MCF-10A morphologies after CYP1B1 induction by CYP1B1 overexpression. (B-C) Cellular migration and invasion were quantified by (B) wound healing assay and (C) transwell invasion assay, respectively, in MCF-10A and MCF-7 cells. Cells were treated with 5 μM DMBA and 10 μM TMS for 48 h. Cells were pre-treated with TMS for 1 h prior to DMBA. (D) MMPs were measured by qPCR following CYP1B1 overexpression in MCF-7 cells. The results were from three independently quantified experiments. (*p≤0.05)

To identify whether CYP1B1 induces EMT-related cell morphology, we observed morphological changes in MCF-10A cells subsequent to CYP1B1 overexpression. In the models of CYP1B1 upregulation, cells acquired mesenchymal morphologies (Fig 2A). To investigate whether CYP1B1 also induces cell migration and invasion, we performed wound healing and transwell invasion assays. In wound healing assays, DMBA-treated MCF-10A cells demonstrated 1.7-fold higher migration rates compared to controls; however, this effect was abrogated when cells were co-treated with DMBA and TMS (Fig 2B). Cell invasion by DMBA-treated MCF-10A cells increased 1.4-fold and DMBA-treated MCF-7 cells increased 1.7-fold compared to controls, but again, this effect was negated in cells treated with both DMBA and TMS (Fig 2C).

Matrix metalloproteinases (MMPs) are established markers of cellular invasion. Therefore, we measured *MMP1*, *MMP9*, *MMP13*, and *MMP14* levels following CYP1B1 modulation and found that CYP1B1 upregulated these MMP transcripts (Fig 2D).

### CYP1B1 activates Wnt/β-catenin signaling pathway

**Fig 3:**
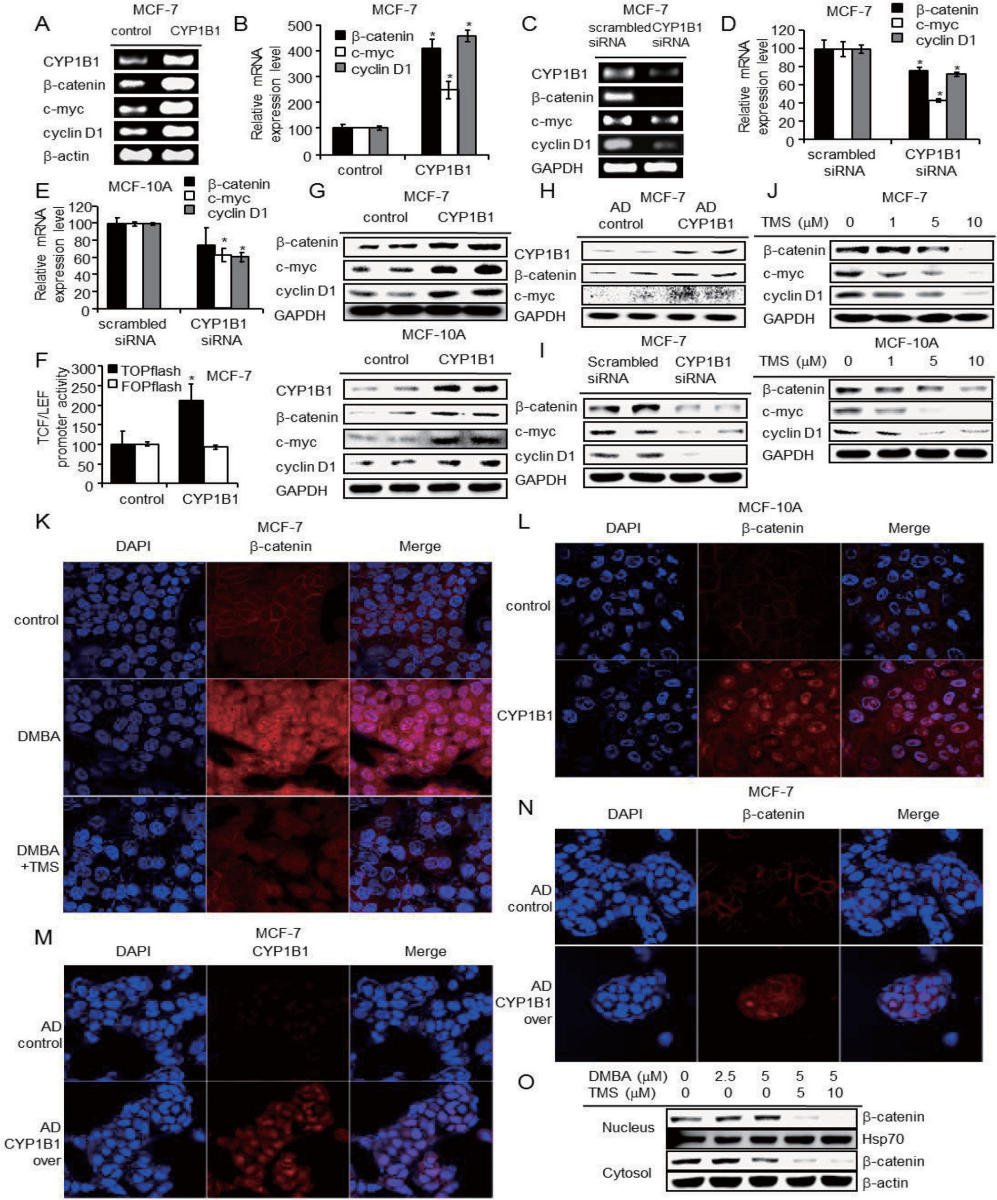
CYP1B1 activates Wnt/β-catenin signaling by inducing β-catenin expression and nuclear localization. (A-B) mRNA expression of β-catenin and Wnt/β-catenin signaling target genes in MCF-7 cells following CYP1B1 overexpression was determined by (A) RT-PCR, (B) qPCR, and (C-D) mRNA expression of β-catenin and Wnt/β-catenin signaling target genes in MCF-7 cells following CYP1B1 knockdown was determined by (C) RT-PCR, and (D) qPCR. (E) mRNA expression of β-catenin and Wnt/β-catenin signaling target genes in MCF-10A cells following CYP1B1 knockdown was determined by qPCR. (F) β-catenin/TCF/LEF promoter activity was determined using dual-luciferase assay following CYP1B1 overexpression in MCF-7 cells. Data are representative of experiments in triplicate. (*p≤0.05) Wnt/β-catenin signaling proteins were measured following (G) CYP1B1 overexpression in MCF-7 and MCF-10A cells, (H) adenoviral CYP1B1 overexpression in MCF-7 cells, (I) CYP1B1 knockdown in MCF-7 cells, and (J) treatment with TMS (0, 1, 5, and 10 μM) for 48 h in MCF-7 and MCF-10A cells. (K) Confocal microscopic analyses of β-catenin following treatment with 5 μM DMBA in the presence of 10 μM TMS for 48 h in MCF-7 cells and (L) CYP1B1 overexpression in MCF-10A cells. (M-N) Confocal microscopic analyses in adenoviral CYP1B1 overexpressed MCF-7 cells for (M) CYP1B1, and (N) β-catenin. (O) β-catenin proteins in nucleus or cytosol were measured following treatment with 5 μM DMBA in the presence of 10 μM TMS for 48 h in MCF-7 cells.

To identify whether CYP1B1 influences Wnt/β-catenin signaling, we measured β-catenin expression after CYP1B1 induction and inhibition. Subsequent to CYP1B1 induction by CYP1B1 overexpression, β-catenin mRNA and protein levels were upregulated while CYP1B1 inhibition accordingly decreased β-catenin expression in MCF-7, MCF-10A, MDA-MB-231, and HeLa cells (Fig 3A–3J; S1 Fig). Confocal microscopic and subcellular fractionation analyses demonstrated that DMBA treatment and CYP1B1 overexpression caused β-catenin to localize to the nucleus, while co-treatment with both DMBA and TMS failed to induce this effect (Fig 3K–3O). CYP1B1 increased mRNA and protein levels of c-Myc and cyclin D1, widely known Wnt/β-catenin target proteins (Fig 3). Furthermore, CYP1B1 enhanced the promoter activity of β-catenin/TCF/LEF (Fig 3F). These results suggest that CYP1B1 promotes cell proliferation via Wnt/β-catenin signaling activation following β-catenin upregulation and nuclear localization.

### CYP1B1 enhances cell invasion through EMT induction

**Fig 4:**
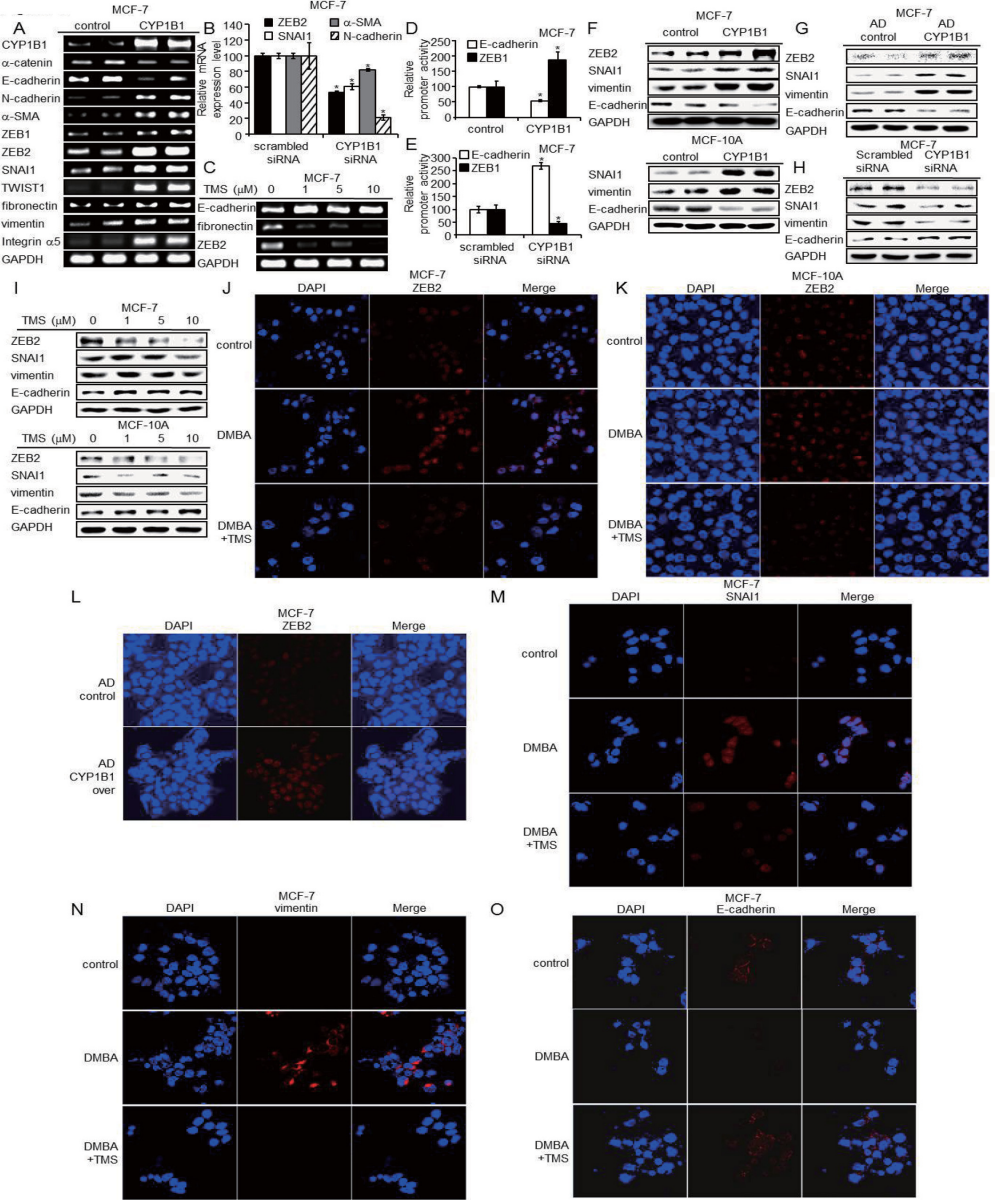
CYP1B1 induces EMT by regulating of EMT-related factors. (A) mRNA expression of EMT-related factors were measured in MCF-7 cells using RT-PCR or qPCR following CYP1B1 overexpression, (B) CYP1B1 knockdown, and (C) TMS treatment (0, 1, 5, and 10 μM) for 48 h. (D-E) ZEB1 and E-cadherin promoter activities in MCF-7 cells were determined by luciferase assay following (D) CYP1B1 overexpression and (E) CYP1B1 knockdown. Data are representative of experiments in triplicate. (*p≤0.05) (F-I) EMT-related factors were measured in MCF-7 and MCF-10A cells using western blot following (F) CYP1B1 overexpression, (G) adenoviral CYP1B1 overexpression, (H) CYP1B1 knockdown, and (I) treatment with TMS (0, 1, 5, and 10 μM) for 48 h. (J-O) The protein levels of EMT-related factors were measured in MCF-7 and MCF-10A cells using confocal microscopy. ZEB2 expression level in (J) 5 μM DMBA and 10 μM TMS treated MCF-7 cells, (K) 5 μM DMBA and 10 μM TMS treated MCF-10A cells, and (L) adenoviral CYP1B1 overexpressed MCF-7 cells. (M) SNAI1, (N) vimentin, and (O) E-cadherin expression in 5 μM DMBA and 10 μM TMS treated MCF-7 cells. As before, cells were pre-treated with TMS for 1 h prior to DMBA.

We observed mesenchymal characteristics in MCF-10A cells with increased CYP1B1 expression (Fig 2A). Generally, the loss of E-cadherin expression during EMT allows cells to break tight junctions and become motile, thus permitting metastasis [32]. To elucidate whether CYP1B1 induces mesenchymal-like phenotypes by initiating EMT, we measured the expression of multiple EMT-related factors in MCF-7 and MCF-10A cells. CYP1B1 induction by overexpression increased mRNA expression of mesenchymal markers including N-cadherin, α-SMA, vimentin, fibronectin, and integrin α5. Transcriptional suppressors of E-cadherin including *ZEB1/2*, *SNAI1*, and *TWIST1* were also induced by CYP1B1. However, CYP1B1 decreased the expression of epithelial markers such as E-cadherin and α-catenin (Fig 4A). These effects were reversed when we decreased CYP1B1 levels by treating cells with TMS or CYP1B1-specific siRNA (Fig 4B and 4C).

*ZEB1* promoter activity was increased in CYP1B1-overexpressing cells and was inhibited in cells following CYP1B1 knockdown, while *CDH1* promoter activity showed the opposite effect (Fig 4D and 4E). We further measured multiple EMT-related factors by western blot, which consistently demonstrated that CYP1B1 induces EMT (Fig 4F–4I). Confocal microscopic analyses of ZEB2, SNAI1, and vimentin also confirmed that CYP1B1 promotes EMT, while these effects were inhibited by TMS (Fig 4J–4N). Furthermore, we found that CYP1B1 considerably decreased E-cadherin expression (Fig 4O).

### CYP1B1-mediated Wnt/β-catenin activation and EMT are regulated by Sp1

**Fig 5:**
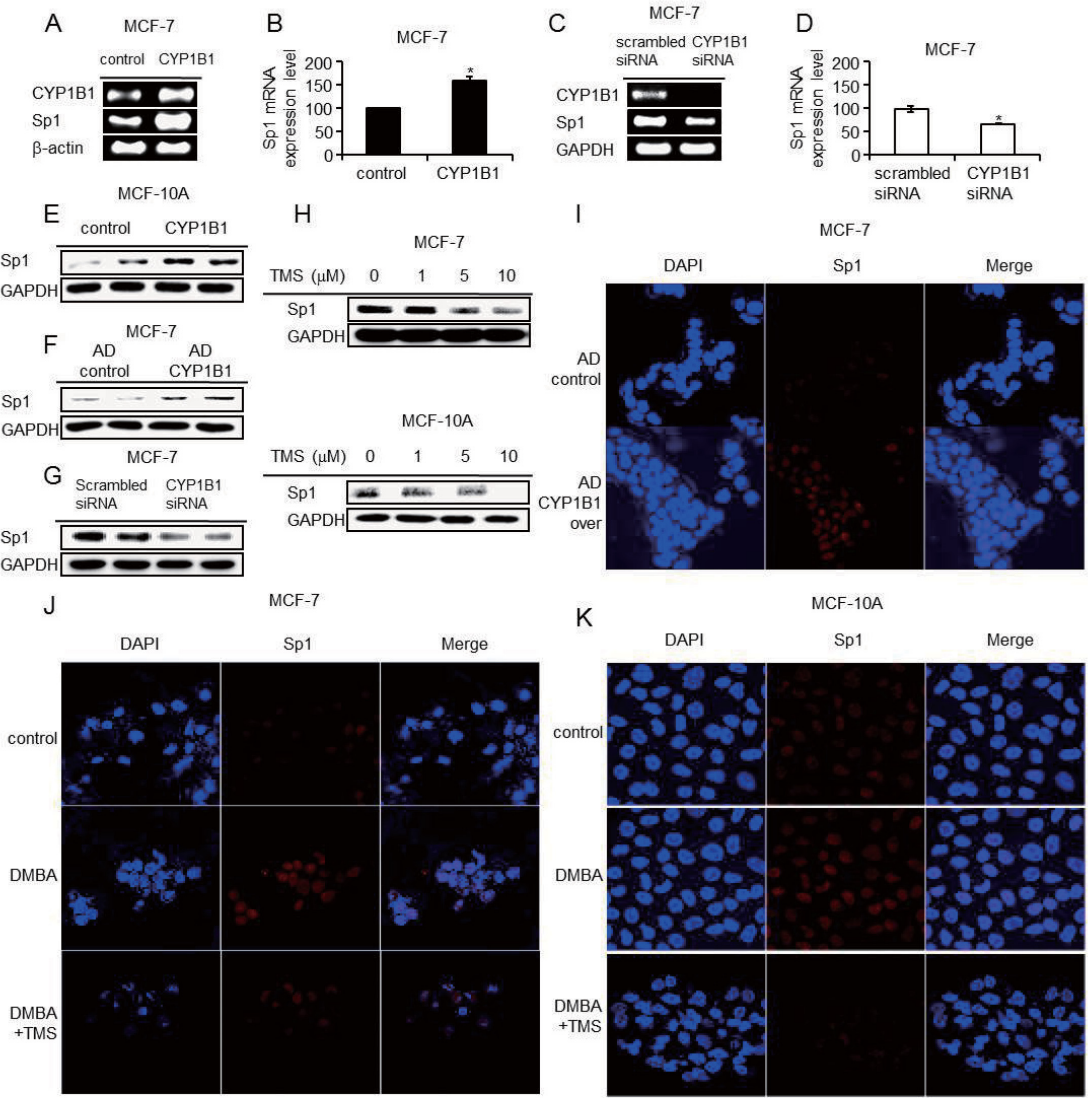
CYP1B1 upregulates Sp1 expression. (A-D) Sp1 mRNA levels were measured in MCF-7 cells by (A) RT-PCR following CYP1B1 overexpression, (B) qRT-PCR following CYP1B1 overexpression, (C) RT-PCR following CYP1B1 knockdown, (D) qRT-PCR following CYP1B1 knockdown. Data are representative of experiments in triplicate. (*p≤0.05) (E-H) Sp1 protein levels were measured by western blot (E) in MCF-10A cells following CYP1B1 overexpression, (F) in MCF-7 cells following adenoviral CYP1B1 overexpression, (G) in MCF-7 cells following CYP1B1 knockdown, and (H) in MCF-7 and MCF-10A cells following TMS treatment (0, 1, 5, and 10 μM) for 48 h. (I-K) Using confocal microscopic analysis, Sp1 levels were determined following (I) adenoviral CYP1B1 overexpression in MCF-7 cells and (J-K) treatment with 5 μM DMBA in the presence of 10 μM TMS for 48 h in (J) MCF-7 and (K) MCF-10A cells. Cells were pre-treated with TMS for 1 h prior to DMBA.

To identify the key regulator of CYP1B1-mediated EMT and Wnt/β-catenin signaling activation, we considered Sp1, because it is widely known as a transcription factor involved in cell proliferation and metastasis. Moreover, Sp1 was recently implicated in ZEB2-induced EMT [33, 34]. Therefore, we investigated whether CYP1B1 regulates Sp1 expression by measuring its expression subsequent to CYP1B1 induction or inhibition in MCF-7, MCF-10A, and MDA-MB-231 cells. Sp1 mRNA and protein was upregulated in CYP1B1-overexpressing cells (Fig 5A, 5B, 5E and 5F). This effect was reversed when CYP1B1 expression was suppressed by TMS or siRNA (Fig 5C, 5D, 5G and 5H; S2 Fig). Confocal microscopic analysis confirmed that CYP1B1 overexpression and DMBA increased Sp1 expression while TMS treatment blocked this effect (Fig 5I–5k). These data indicate that CYP1B1 positively regulates Sp1 expression.

**Fig 6:**
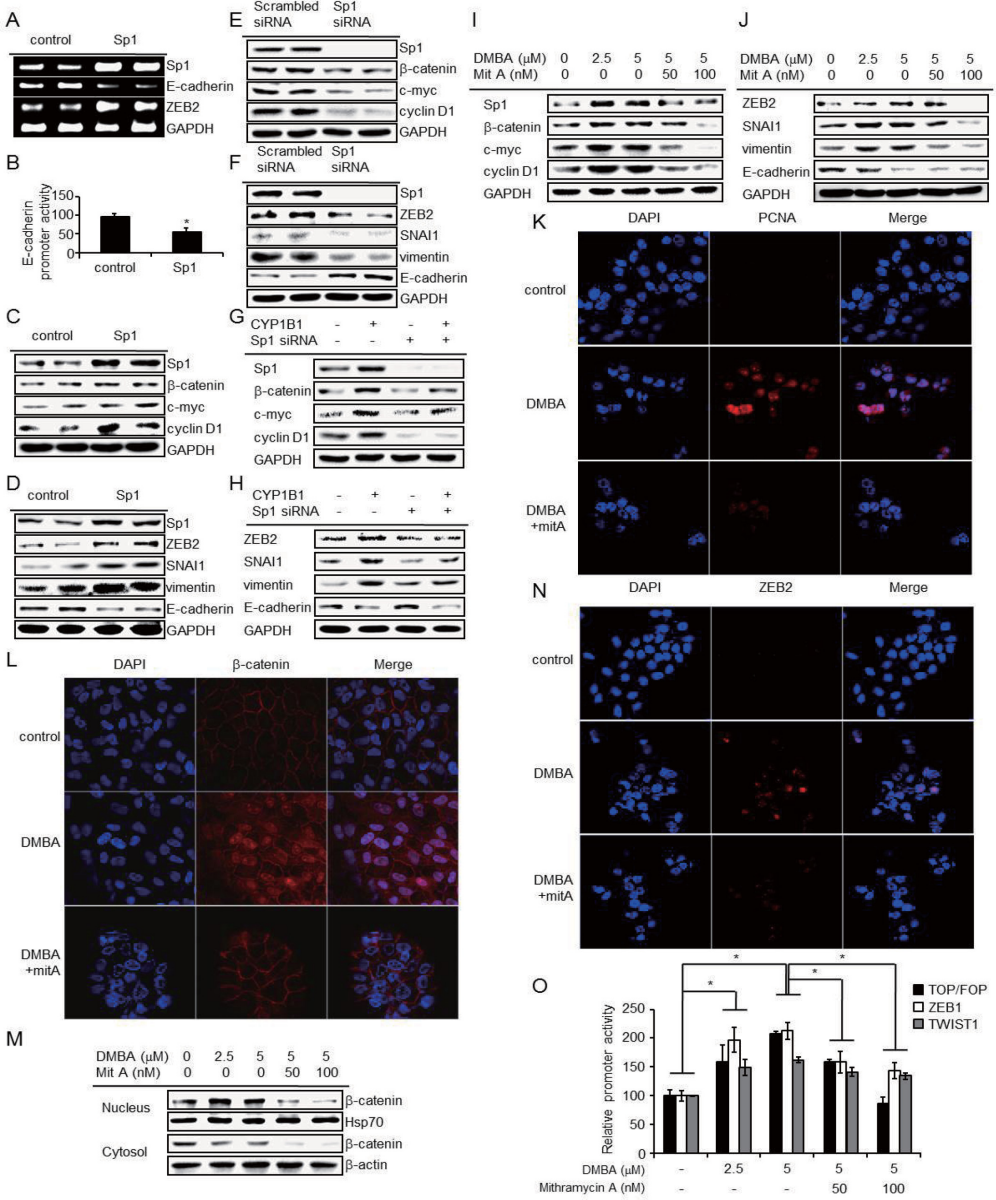
Sp1 is sufficient to induce CYP1B1-mediated effects. (A) ZEB2 and E-cadherin mRNA were measured by RT-PCR and (B) E-cadherin promoter activity was determined by luciferase assay after Sp1 overexpression in MCF-7 cells. (C) Key proteins in Wnt/β-catenin signaling and (D) EMT-related factors were measured by western blot after Sp1 induction in MCF-7 cells. (E and F) Similar to Fig 5C and 5D, but following Sp1 inhibition. (G and H) Also similar to Fig 5C and 5D, but in MCF-7 cells co-transfected with CYP1B1 overexpression vector and Sp1 siRNA. (I and J) MCF-7 cells treated with DMBA and mithramycin A for 24 h following pre-treatment with mithramycin A for 1 h. (I) Key factors in Wnt/β-catenin signaling and (J) EMT were determined by western blot. (K) PCNA in MCF-7 cells, (L) β-catenin in MCF-10A cells, (N) ZEB2 in MCF-7 cells were observed by confocal microscopy after treatment with 5 μM DMBA and 100 nM mithramycin A for 24 h. (M) β-catenin proteins in nuclear or cytosolic fraction of MCF-10A cells were measured following treatment with 5 μM DMBA and 100 nM mithramycin A for 24 h. Cells were pre-treated with mithramycin A for 1 h prior to DMBA. (O) Relative promoter activity of β-catenin/TCF/LEF, ZEB1, and TWIST1 was determined by dual-luciferase assay after treatment with DMBA and mithramycin A. As before, cells were pre-treated with mithramycin A for 1 h prior to DMBA. Data are representative of experiments in triplicate. (*p≤0.05)

Next, we investigated whether Sp1 modulates the key regulators involved in EMT and Wnt/β-catenin signaling and found that Sp1 upregulates these pathways (Fig 6A–6F). Specifically, *CDH1* promoter activity in Sp1-overexpressing cells was 40% as active compared to control cells (Fig 6B). To ascertain whether Sp1 is required for CYP1B1-mediated effects, we measured the expression of β-catenin, c-Myc, cyclin D1, ZEB2, SNAI1, and vimentin in cells with CYB1B1 overexpression and Sp1 knockdown. Sp1 knockdown prevented CYP1B1-mediated Wnt/β-catenin activation (Fig 6G; S3A Fig). Moreover, ZEB2, SNAI1, and vimentin levels normally induced by CYP1B1 were markedly suppressed in Sp1 knockdown cells (Fig 6H; S3B Fig).

To clarify whether Sp1 DNA-binding plays a role in CYP1B1-mediated transcriptional regulation, we co-treated cells with DMBA and mithramycin A, an inhibitor of Sp1 DNA-binding, and measured the expression of multiple key proteins. The upregulation of β-catenin, c-Myc, cyclin D1, ZEB2, vimentin, and SNAI1 induced by DMBA was suppressed by mithramycin A in a concentration-dependent manner (Fig 6I and 6J; S4 Fig). These data suggested that Sp1 serves as the transcription factor that facilitates CYP1B1-mediated oncogenesis.

In confocal microscopic and subcellular fractionation analyses, DMBA likewise increased PCNA, ZEB2, and β-catenin. Interestingly, when cells were treated with both DMBA and mithramycin A (100 nM), the induction of PCNA, ZEB2, and β-catenin was almost completely blocked and β-catenin failed to localize to the nucleus (Fig 6K–6N). Similarly, the enhanced promoter activities of β-catenin/TCF/LEF, ZEB1, and TWIST1observed with DMBA treatment were suppressed in the presence of mithramycin A (Fig 6O). These results suggest that Sp1 directly regulates the transcriptional activities of β-catenin, ZEB1, and TWIST1 and initiates EMT and Wnt/β-catenin signaling.

### 4-Hydroxyestradiol (4-OHE_2_) may play an important role in CYP1B1-mediated oncogenesis

**Fig 7:**
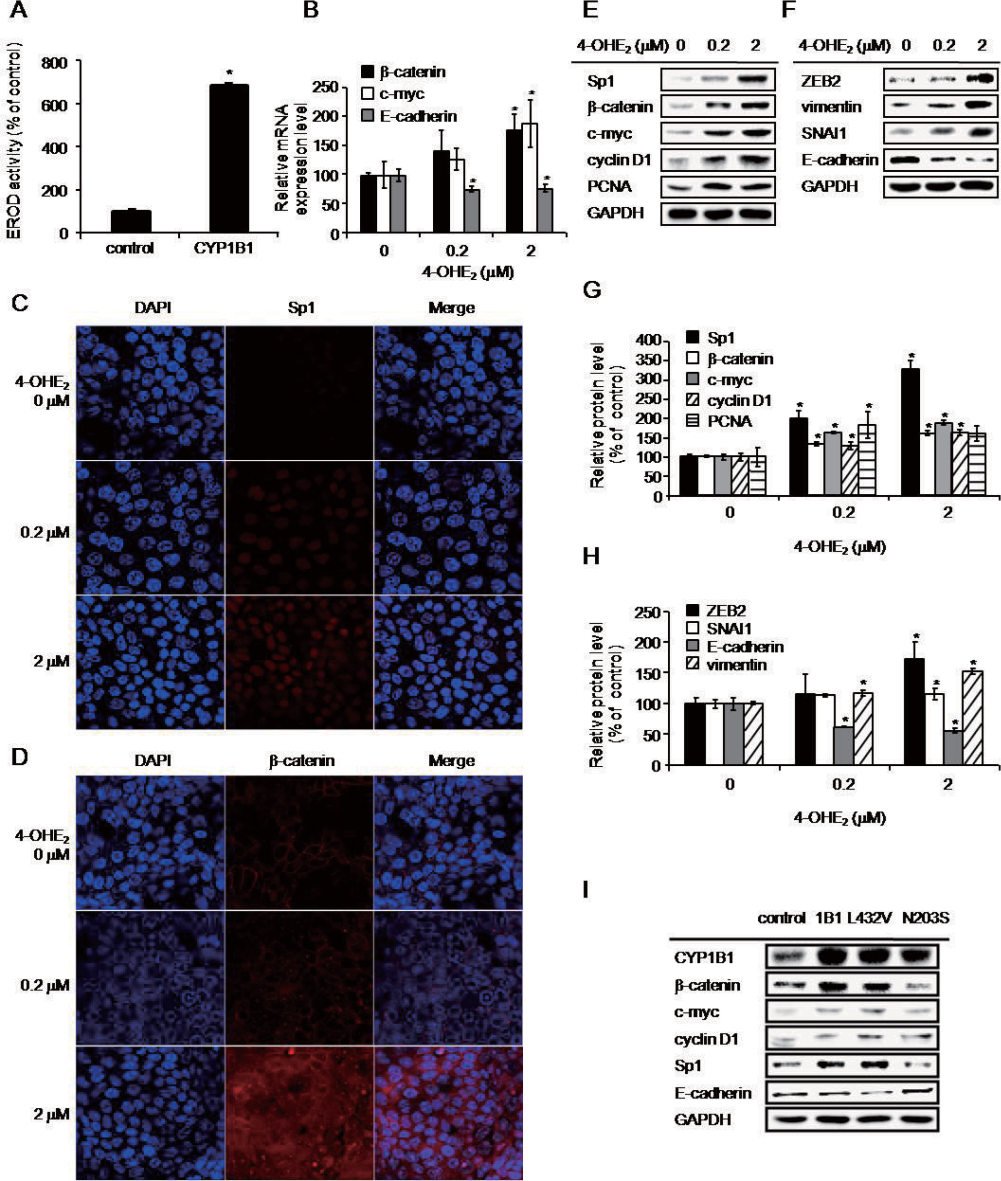
4-OHE_2_ induces CYP1B1-mediated oncogenic events through upregulation of Sp1. (A) Enzyme activity of CYP1B1 was determined by EROD assay in CYP1B1-overexpressed MCF-7 cells. Data are representative of experiments in duplicate. (*p≤0.05) (B) Wnt/β-catenin signaling target genes and E-cadherin mRNA were measured by qPCR and (C) Sp1 and (D) β-catenin expression were analyzed by confocal microscopy in 4-OHE_2_-treated MCF-7 cells. (E) Protein levels of Wnt/β-catenin signaling target proteins and (F) EMT-related factors were determined using western blot in 4-OHE_2_-treated MCF-7 cells. All western blots were performed independently three times and the bands were quantified using Quantity One software program. (G) Wnt/β-catenin signaling target proteins in 4-OHE_2_-treated cells, and (H) EMT-related factors in 4-OHE_2_-treated cells. The results were from three independently quantified experiments. (*p≤0.05) (I) Wnt/β-catenin signaling target proteins, Sp1, and E-cadherin proteins were measured by western blot following overexpression of CYP1B1 polymorphic genes in MCF-10A cells.

To clarify whether CYP1B1-mediated EMT and Wnt/β-catenin signaling activation are initiated by CYP1B1 activity, we examined the enzyme activity of CYP1B1 following CYP1B1 overexpression (Fig 7A). The significant increase of CYP1B1 enzymatic activity could suggest that the oncogenic events occurred by CYP1B1 overexpression may be the results of CYP1B1 activity. To identify whether our hypothesis is valid, the expression levels of key proteins following treatment of the enzymatic products of CYP1B1, 4-OHE_2_ or 2-OHE_2_. *CTNNB1* and *MYC* mRNA levels were upregulated whereas *CDH1* was suppressed in 4-OHE_2_-treated cells (Fig 7B). Sp1, a key regulator for CYP1B1-mediated effects, was induced by 4-OHE_2_ in a concentration-dependent manner (Fig 7C). β-catenin protein also increased with 4-OHE_2_ treatment, and we found that β-catenin in 4-OHE_2_-treated cells localized to the nucleus, as was observed in DMBA-treated or CYP1B1-overexpressing cells (Fig 7D). To compare the effects of estrogen metabolites produced by CYP1B1, cells were treated with 4-OHE_2_ or 2-OHE_2_ (Fig 7E and 7F; S5A and S5B Fig). 4-OHE_2_ significantly increased Sp1, β-catenin, c-Myc, cyclin D1, PCNA, ZEB2, SNAI1, and vimentin expression and decreased E-cadherin levels, while 2-OHE_2_ did not demonstrate any significant effects (Fig 7G and 7H; S5C Fig). The allelic variants of CYP1B1 gene having higher or lower enzymatic activity have been reported previously and CYP1B1 L432V and N203S have been reported to have markedly higher and lower enzymatic activity, respectively [35–37]. To elucidate whether the enzymatic activity of CYP1B1 is a major cause of EMT induction and Wnt/β-catenin signaling activation, the expression levels of Wnt/β-catenin signaling target proteins and Sp1 were determined following overexpression of CYP1B1 L432V or N203S polymorphic genes and showed to be positively regulated by CYP1B1 enzymatic activity. E-cadherin, however, showed the opposite result (Fig 7I). These data indicate that the activity of CYP1B1 with generation of 4-OHE_2_, a major metabolite produced from estrogen by CYP1B1, may play a crucial role in CYP1B1-mediated EMT and Wnt/β-catenin signaling activation through induction of Sp1.

## Discussion

Increased cell proliferation, migration, and invasion are widely considered as cancer hallmarks and key processes for tumor progression. To the best of our knowledge, the current study represents the first evidence that CYP1B1 enhances EMT and activates Wnt/β-catenin signaling by upregulating Sp1. Sp1 expression was promoted in cells treated with 4-OHE_2_ and mediated the upregulation of EMT-inducing factors. This cascade of events inhibited E-cadherin expression and simultaneously increased Wnt/β-catenin signaling through the upregulation and nuclear localization of β-catenin. These results demonstrate that Sp1 mediates the downstream transcriptional effects associated with elevated CYP1B1 and is essential for EMT and Wnt/β-catenin signaling.

Up to this point, the relationship between Sp1 and Wnt/β-catenin signaling has been unclear. Several studies have reported that Sp1-related transcription factors might act as activators of Wnt/β-catenin target genes during cell development [38, 39]. Importantly, we show that CYP1B1-induced cell proliferation in MCF-7 and MCF-10A cells is caused by PCNA upregulation. PCNA acts as an auxiliary component of the DNA polymerase-δ complex and plays an important role in DNA replication [40]. Recently, the relationship between PCNA and Wnt/β-catenin signaling became clearer with the report that PAF (PCNA-associated factor) can dissociate from PCNA complexes and bind to β-catenin, which enhances Wnt/β-catenin target gene expression upon Wnt signaling activation [41]. Based on these data, we suggest that PCNA mediates CYP1B1-induced Wnt/β-catenin signal activation, and that the relationship between PCNA and Sp1 be investigated in detail.

In this study, we found that Sp1 upregulates E-cadherin repressors like ZEB1/2, SNAIL, and TWIST1, which subsequently induce EMT. Recently, it has been reported that Sp1 induces cell migration and invasion in cooperation with ZEB2 [34]. Moreover, Sp1 has been shown to inhibit miR-200a expression; this subsequently allows HDAC4-mediated promoter diacetylation at *ZEB1/2*, which inhibits their expression [42, 43]. The relationship between Sp1 and SNAIL is not fully understood, although it has been shown that Sp1 directly binds to the *SNAIL* promoter and thus upregulates SNAIL during EMT [44]. Moreover, SNAIL can induce Sp1 by suppressing an inhibitor of Sp1, miR-128 [45]. These data suggest that Sp1 and SNAIL mutually upregulate one another. Finally, Sp1 upregulation of *TWIST1* expression by associating with CCT repeats in the *TWIST1* promoter has been suggested; however, this process requires further investigation [46].

During invasion, cancer cells secrete MMPs to induce extracellular matrix (ECM) degradation. Sp1 has been reported to regulate the expression of multiple MMPs. For example, the promoters of *MMP1*, *MMP9*, and *MMP14* contain Sp1 binding sites, and transcriptions from these loci are directly upregulated by Sp1 [47, 48]. MMP1 and MMP14 have been reported to induce cancer cell invasion, and MMP14 is further recognized in the activation of MMP2 and MMP9 [49, 50]. MMP13 expression is also increased by Sp1, and both MMP13 and MMP9 are implicated in the progression of various tumors [51–54]. In this study, we show that CYP1B1 upregulated the transcripts for all of these MMPs. Therefore, Sp1 is likewise assumed to play an important role in cell invasion, since CYP1B1 increases Sp1 expression and DNA binding.

There are several studies that have been reported the effects of CYP1B1 knockout *in vivo* models. The *Cyp1b1*(-/-) mice represented the elevated protection against DNA adduct formation induced by carcinogenic agents like DMBA or benzo[a,l]pyrene in tumors [55–57] and also showed the blocking effect on tumor tissue metastasis induced by benzo[a]pyrene [58]. Based on these previously reported *in vivo* data, the novel mechanism of CYP1B1-induced cell proliferation, migration, and invasion might have the preclinical significance but the *in vivo* experiments such as transplantation assay should be investigated in further study.

Although CYP1B1 upregulation via Sp1 binding in the *CYP1B1* promoter has been reported, the reciprocal effect of CYP1B1 on Sp1 expression has not yet been described [59]. Recently, estrogens have been reported to regulate microRNA expression [60]. Among the estrogen-dependent microRNAs, miR-375 is generally suppressed in multiple cancers, including gastric, cervical, liver, lung, and esophageal cancer. This downregulation has recently been attributed to hypermethylation of its promoter in cancer cells [61–65]. These findings suggest that miR-375 may act as a tumor suppressor. As miR-375 directly binds the 3′UTR of Sp1 and thereby negatively regulates Sp1 expression, this microRNA might suppress cell migration and invasion [61]. Furthermore, miR-375 downregulation accompanies tamoxifen resistance and EMT in tamoxifen-resistant breast cancer cells [66]. Since CYP1B1 overexpression and 4-OHE_2_ treatment induce Sp1 expression, 4-OHE_2_ might be responsible for the suppression of miR-375 or other microRNAs, which subsequently promotes Sp1 expression.

**Fig 8:**
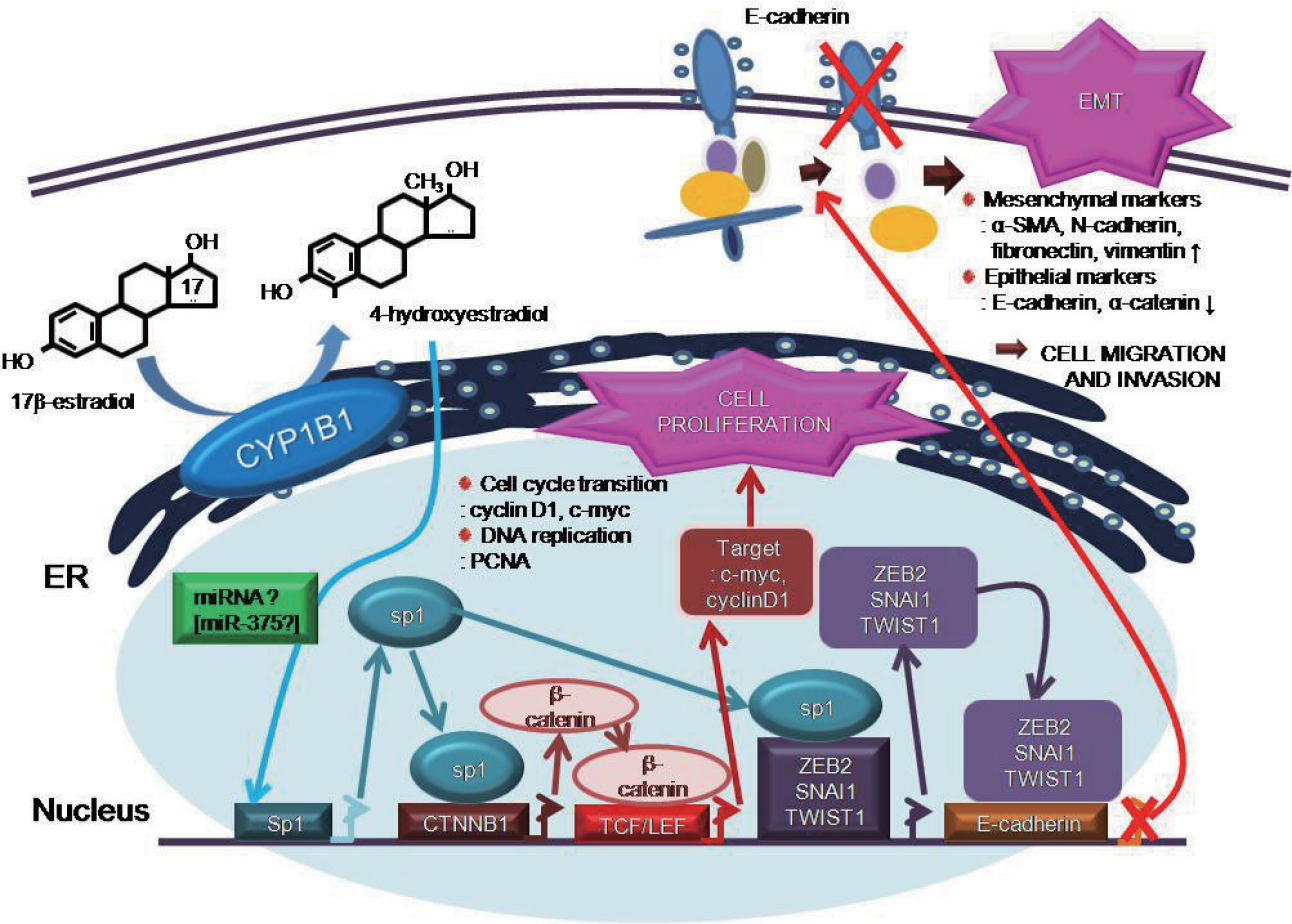
Scheme for the novel mechanisms of CYP1B1 action. Scheme for CYP1B1-induced cell proliferation via activation of Wnt/β-catenin signaling and CYP1B1-induced celll migration and invasion through induction of EMT.

In summary, to the best of our knowledge, our present study is the first report to identify the molecular mechanism underlying CYP1B1-mediated cancer progression. Our results demonstrate that CYP1B1 enhances cell proliferation via Wnt/β-catenin signaling activation by inducing the expression and nuclear localization of β-catenin. Moreover, EMT induction by CYP1B1 was mediated by the upregulation of E-cadherin transcriptional repressors. Our results further indicate that CYP1B1 enzymatic activity is essential for CYP1B1-mediated EMT and Wnt/β-catenin signaling activation, because 4-OHE_2_ treatment was sufficient to induce Sp1 and other key proteins in EMT and Wnt/β-catenin signaling. The scheme in Fig 8 summarizes these novel findings revealing CYP1B1-induced oncogenic mechanisms. Since CYP1B1 is implicated as a significant factor in the development of various cancers, a more detailed understanding of the precise mechanisms underpinning CYP1B1-mediated cancer progression may facilitate the development of new strategies for cancer treatment.

## Supporting Information

S1 Fig CYP1B1 upregulates Wnt/β-catenin signaling target proteins in MDA-MB-231 and HeLa cells. (A) β-catenin protein levels were measured following treatment with TMS (0, 1, 5, and 10 μM) for 48 h in MDA-MB-231 cells, and (B) CYP1B1 overexpression in HeLa cells.
(EPS)

S2 Fig CYP1B1 upregulates Sp1 expression in MDA-MB-231 cells. (A) Sp1 mRNA and protein levels were measured in MDA-MB-231 cells by RT-PCR following TMS treatment (0, 1, 5, and 10 μM) for 48 h, (B) by western blot following TMS treatment (0, 1, 5, and 10 μM) for 48 h.
(EPS)

S3 Fig Sp1 is sufficient to induce CYP1B1-mediated effects. (A) Key proteins in Wnt/β-catenin signaling and (B) EMT-related factors were measured by western blot after MCF-10A cells were co-transfected with CYP1B1 overexpression vector and Sp1 siRNA.
(EPS)

S4 Fig DNA binding ability of Sp1 is sufficient to induce CYP1B1-mediated effects. MCF-10A cells treated with 5 μM DMBA for 24 h following pre-treatment with 100 nM mithramycin A for 1 h. (A) Protein levels of Wnt/β-catenin signaling target genes and (B) EMT-related factors were determined using western blot.
(EPS)

S5 Fig 2-OHE_2_ has no significant effect on CYP1B1-mediated oncogenic events. (A) Wnt/β-catenin signaling target proteins and (B) EMT-related factors in 2-OHE_2_-treated MCF-7 cells were measured by western blot. All western blots were performed independently three times and the bands were quantified using Quantity One software program. (C) Wnt/β-catenin signaling target proteins in 2-OHE_2_-treated MCF-7 cells and (D) EMT-related factors in 2-OHE_2_-treated MCF-7 cells. The results were from three independently quantified experiments. (*p≤0.05)
(EPS)

S1 Table Primers for quantitative realtime-PCR (qPCR) analysis. (DOCX)

## Abbreviations

CYP1B1: Cytochrome P450 1B1
DMBA: 7,12-dimethylbenz[α]anthracene
TMS: Tetramethoxystilbene
EMT: Epithelial-mesenchymal transition
OHE_2_: hydroxyestradio
CTNNB1: β-catenin (gene
DAPI: 4′,6-diamidino-2-phenylindole
Sp1: Specificity protein 1
ZEB: Zinc finger E-box binding homeobox
MMP: Matrix metalloproteinase
GAPDH: Glyceraldehyde 3-phosphate dehydrogenase
Hsp: Heat shock protein
PMSF: Phenylmethanesulfonylfluoride
BCA: Bicinchoninic acid
SDS-PAGE: Sodium dodecyl sulfate-polyacrylamide gel electrophoresis
PVDF: Polyvinylidene fluoride
PBS: Phosphate buffered saline
PCNA: Proliferating Cell Nuclear Antigen
α-SMA: α-Smooth Muscle Actin
CDH1: E-cadherin (gene)
miR: microRNA
HDAC: Histone deacetylase
PAF: PCNA-associated facto

## References

[1] Sutter TR, Tang YM, Hayes CL, Wo YY, Jabs EW, Li X, et al Complete cDNA sequence of a human dioxin-inducible mRNA identifies a new gene subfamily of cytochrome P450 that maps to chromosome 2. J Biol Chem 1994;269:13092–13099. 8175734

[2] Spink DC, Hayes CL, Young NR, Christou M, Sutter TR, Jefcoate CR, et al The effects of 2,3,7,8-tetrachlorodibenzo-p-dioxin on estrogen metabolism in MCF-7 breast cancer cells: evidence for induction of a novel 17β-estradiol 4-hydroxylase. J Steroid Biochem Mol Biol 1994;51:251–258. 7826886 10.1016/0960-0760(94)90037-x

[3] Belous AR, Hachey DL, Dawling S, Roodi N, Parl FF. Cytochrome P450 1B1-mediated estrogen metabolism results in estrogen-deoxyribonucleoside adduct formation. Cancer Res 2007;67:812–817. 17234793 10.1158/0008-5472.CAN-06-2133

[4] Hachey DL, Dawling S, Roodi N, Parl FF. Sequential action of phase I and II enzymes cytochrome p450 1B1 and glutathione S-transferase P1 in mammary estrogen metabolism. Cancer Res 2003;63:8492–8499. 14679015

[5] Tang YM, Wo YY, Stewart J, Hawkins AL, Griffin CA, Sutter TR, et al Isolation and characterization of the human cytochrome P450 CYP1B1 gene. J Biol Chem 1996;271:28324–28330. 8910454 10.1074/jbc.271.45.28324

[6] Shimada T, Hayes CL, Yamazaki H, Amin S, Hecht SS, Guengerich FP, et al Activation of chemically diverse procarcinogens by human cytochrome P-450 1B1. Cancer Res 1996;56:2979–2984. 8674051

[7] Murray GI, Taylor MC, McFadyen MC, McKay JA, Greenlee WF, Burke MD, et al Tumor-specific expression of cytochrome P450 CYP1B1. Cancer Res 1997;57:3026–3031. 9230218

[8] Patel SA, Gooderham NJ. Interleukin-6 promotes dietary carcinogen-induced DNA damage in colorectal cancer cells. Toxicol Res 2015;4:858–866.

[9] McKay JA, Melvin WT, Ah-See AK, Ewen SW, Greenlee WF, Marcus CB, et al Expression of cytochrome P450 CYP1B1 in breast cancer. FEBS Lett 1995;374:270–272. 7589551 10.1016/0014-5793(95)01126-y

[10] Tokizane T, Shiina H, Igawa M, Enokida H, Urakami S, Kawakami T, et al Cytochrome P450 1B1 is overexpressed and regulated by hypomethylation in prostate cancer. Clin Cancer Res 2005;11:5793–5801. 16115918 10.1158/1078-0432.CCR-04-2545

[11] McFadyen MC, Cruickshank ME, Miller ID, McLeod HL, Melvin WT, Haites NE, et al Cytochrome P450 CYP1B1 over-expression in primary and metastatic ovarian cancer. Br J Cancer 2001;85:242–246. 11461084 10.1054/bjoc.2001.1907 PMC2364045

[12] Ragavan N, Hewitt R, Cooper LJ, Ashton KM, Hindley AC, Nicholson CM, et al CYP1B1 expression in prostate is higher in the peripheral than in the transition zone. Cancer Lett 2004;215:69–78. 15374634 10.1016/j.canlet.2004.06.051

[13] Singh PB, Ragavan N, Ashton KM, Basu P, Nadeem SM, Nicholson CM, et al Quantified gene expression levels for phase I/II metabolizing enzyme and estrogen receptor levels in benign prostate from cohorts designated as high-risk (UK) versus low-risk (India) for adenocarcinoma at this organ site: a preliminary study. Asian J Androl 2010;12:203–214. 10.1038/aja.2009.71 19935673 PMC3739086

[14] Le Marchand L, Donlon T, Kolonel LN, Henderson BE, Wilkens LR. Estrogen metabolism-related genes and breast cancer risk: the multiethnic cohort study. Cancer Epidemiol Biomarkers Prev 2005;14:1998–2003. 16103451 10.1158/1055-9965.EPI-05-0076

[15] Saini S, Hirata H, Majid S, Dahiya R. Functional significance of cytochrome P450 1B1 in endometrial carcinogenesis. Cancer Res 2009;69:7038–7045. 10.1158/0008-5472.CAN-09-1691 19690133

[16] Hong M, Park N, Chun YJ. Role of annexin a5 on mitochondria-dependent apoptosis induced by tetramethoxystilbene in human breast cancer cells. Biomol Ther 2014;22:519–524 10.4062/biomolther.2014.112 PMC4256031 25489419

[17] Chen B, Qiu LX, Li Y, Xu W, Wang XL, Zhao WH, et al The CYP1B1 Leu432Val polymorphism contributes to lung cancer risk: evidence from 6501 subjects. Lung Cancer 2010;70:247–252. 10.1016/j.lungcan.2010.03.011 20395011

[18] Jiang W, Sun G, Xiong J, Xi X, Shi Z. Association of CYP1B1 L432V polymorphism with urinary cancer susceptibility: a meta-analysis. Diagn Pathol 2014;9:113 10.1186/1746-1596-9-113 24913727 PMC4067118

[19] De Vivo I, Hankinson SE, Li L, Colditz GA, Hunter DJ. Association of CYP1B1 polymorphisms and breast cancer risk. Cancer Epidemiol Biomarkers Prev 2002;11:489–492. 12010864

[20] Polakis P. Wnt signaling and cancer. Genes Dev 2000;14:1837–1851. 10921899

[21] Giles RH, van Es JH, Clevers H. Caught up in a Wnt storm: Wnt signaling in cancer. Biochim Biophys Acta 2003;1653:1–24. 12781368 10.1016/s0304-419x(03)00005-2

[22] Aberle H, Schwartz H, Kemler R. Cadherin-catenin complex: protein interactions and their implications for cadherin function. J Cell Biochem 1996;61:514–523. 8806074 10.1002/(SICI)1097-4644(19960616)61:4%3C514::AID-JCB4%3E3.0.CO;2-R

[23] Son H, Moon A. Epithelial-mesenchymal transition and cell invasion. Toxicol Res 2010;26:245–252. 10.5487/TR.2010.26.4.245 24278531 PMC3834497

[24] Carvell MJ, Marsh PJ, Persaud SJ, Jones PM. E-cadherin interactions regulate beta-cell proliferation in islet-like structures. Cell Physiol Biochem 2007;20:617–626. 17762188 10.1159/000107545

[25] Batlle E, Sancho E, Franci C, Dominguez D, Monfar M, Baulida J, et al The transcription factor snail is a repressor of E-cadherin gene expression in epithelial tumour cells. Nat Cell Biol 2000;2:84–89. 10655587 10.1038/35000034

[26] Comijn J, Berx G, Vermassen P, Verschueren K, van Grunsven L, Bruyneel E, et al The two-handed E box binding zinc finger protein SIP1 downregulates E-cadherin and induces invasion. Mol Cell 2001;7:1267–1278. 11430829 10.1016/s1097-2765(01)00260-x

[27] Thiery JP. Epithelial-mesenchymal transitions in tumour progression. Nat Rev Cancer 2002;2:442–454. 12189386 10.1038/nrc822

[28] Ciruna B, Rossant J. FGF signaling regulates mesoderm cell fate specification and morphogenetic movement at the primitive streak. Dev Cell 2001;1:37–49. 11703922 10.1016/s1534-5807(01)00017-x

[29] Onder TT, Gupta PB, Mani SA, Yang J, Lander ES, Weinberg RA. Loss of E-cadherin promotes metastasis via multiple downstream transcriptional pathways. Cancer Res 2008;68:3645–3654. 10.1158/0008-5472.CAN-07-2938 18483246

[30] Maga G, Hubscher U. Proliferating cell nuclear antigen (PCNA): a dancer with many partners. J Cell Sci 2003;116:3051–3060. 12829735 10.1242/jcs.00653

[31] Debnath J, Muthuswamy SK, Brugge JS. Morphogenesis and oncogenesis of MCF-10A mammary epithelial acini grown in three-dimensional basement membrane cultures. Methods 2003;30:256–268. 12798140 10.1016/s1046-2023(03)00032-x

[32] Christiansen JJ, Rajasekaran AK. Reassessing epithelial to mesenchymal transition as a prerequisite for carcinoma invasion and metastasis. Cancer Res 2006;66:8319–8326. 16951136 10.1158/0008-5472.CAN-06-0410

[33] Zhang JP, Zhang H, Wang HB, Li YX, Liu GH, Xing S, et al Down-regulation of Sp1 suppresses cell proliferation, clonogenicity and the expressions of stem cell markers in nasopharyngeal carcinoma. J Transl Med 2014;12:222 10.1186/s12967-014-0222-1 25099028 PMC4132216

[34] Nam EH, Lee Y, Zhao XF, Park YK, Lee JW, Kim S. ZEB2-Sp1 cooperation induces invasion by upregulating cadherin-11 and integrin α5 expression. Carcinogenesis 2014;35:302–314. 10.1093/carcin/bgt340 24130169

[35] Li DN, Seidel A, Pritchard MP, Wolf CR, Friedberg T. Polymorphisms in P450 CYP1B1 affect the conversion of estradiol to the potentially carcinogenic metabolite 4-hydroxyestradiol. Pharmacogenetics 2000;10:343–353. 10862525 10.1097/00008571-200006000-00008

[36] Chavarria-Soley G, Sticht H, Aklillu E, Ingelman-Sundberg M, Pasutto F, Reis A, Rautenstrauss B. Mutations in CYP1B1 cause primary congenital glaucoma by reduction of either activity or abundance of the enzyme. Hum Mutat 2008;29:1147–1153. 10.1002/humu.20786 18470941

[37] Campos-Mollo E, Lopez-Garrido MP, Blanco-Marchite C, Garcia-Feijoo J, Peralta J, Belmonte-Martinez J, et al CYP1B1 mutations in Spanish patients with primary congenital glaucoma: phenotypic and functional variability. Mol Vis 2009;15:417–431. 19234632 PMC2645906

[38] Weidinger G, Thorpe CJ, Wuennenberg-Stapleton K, Ngai J, Moon RT. The Sp1-related transcription factors sp5 and sp5-like act downstream of Wnt/beta-catenin signaling in mesoderm and neuroectoderm patterning. Curr Biol 2005;15:489–500. 15797017 10.1016/j.cub.2005.01.041

[39] Thorpe CJ, Weidinger G, Moon RT. Wnt/β-catenin regulation of the Sp1-related transcription factor sp5l promotes tail development in zebrafish. Development 2005;132:1763–1772. 15772132 10.1242/dev.01733

[40] Bravo R, Frank R, Blundell PA, Macdonald-Bravo H. Cyclin/PCNA is the auxiliary protein of DNA polymerase-δ. Nature 1987;326:515–517. 2882423 10.1038/326515a0

[41] Jung HY, Jun S, Lee M, Kim HC, Wang X, Ji H, et al PAF and EZH2 induce Wnt/β-catenin signaling hyperactivation. Mol Cell 2013;52:193–205. 10.1016/j.molcel.2013.08.028 24055345 PMC4040269

[42] Wang G, Guo X, Hong W, Liu Q, Wei T, Lu C, et al Critical regulation of miR-200/ZEB2 pathway in Oct4/Sox2-induced mesenchymal-to-epithelial transition and induced pluripotent stem cell generation. Proc Natl Acad Sci U SA 2013;110:2858–2863. 10.1073/pnas.1212769110 PMC3581874 23386720

[43] Yuan JH, Yang F, Chen BF, Lu Z, Huo XS, Zhou WP, et al The histone deacetylase 4/SP1/microrna-200a regulatory network contributes to aberrant histone acetylation in hepatocellular carcinoma. Hepatology 2011;54:2025–2035. 10.1002/hep.24606 21837748

[44] Barbera MJ, Puig I, Dominguez D, Julien-Grille S, Guaita-Esteruelas S, Peiro S, et al Regulation of Snail transcription during epithelial to mesenchymal transition of tumor cells. Oncogene 2004;23:7345–7354. 15286702 10.1038/sj.onc.1207990

[45] Dong Q, Cai N, Tao T, Zhang R, Yan W, Li R, et al An axis involving SNAI1, microRNA-128 and SP1 modulates glioma progression. PLoS One 2014;9:e98651 10.1371/journal.pone.0098651 24959930 PMC4068992

[46] Ohkuma M, Funato N, Higashihori N, Murakami M, Ohyama K, Nakamura M. Unique CCT repeats mediate transcription of the TWIST1 gene in mesenchymal cell lines. Biochem Biophys Res Commun 2007;352:925–931. 17157810 10.1016/j.bbrc.2006.11.114

[47] Poplineau M, Schnekenburger M, Dufer J, Kosciarz A, Brassart-Pasco S, Antonicelli F, et al The DNA hypomethylating agent, 5-aza-2'-deoxycytidine, enhances tumor cell invasion through a transcription-dependent modulation of MMP-1 expression in human fibrosarcoma cells. Mol Carcinog 2015;54:24–34. 10.1002/mc.22071 24038389

[48] Turner NA, Porter KE. Regulation of myocardial matrix metalloproteinase expression and activity by cardiac fibroblasts. IUBMB Life 2012;64:143–150. 10.1002/iub.594 22215527

[49] Foley CJ, Luo C, O'Callaghan K, Hinds PW, Covic L, Kuliopulos A. Matrix metalloprotease-1a promotes tumorigenesis and metastasis. J Biol Chem 2012;287:24330–24338. 10.1074/jbc.M112.356303 22573325 PMC3397859

[50] Hernandez-Perez M, El-hajahmad M, Massaro J, Mahalingam M. Expression of gelatinases (MMP-2, MMP-9) and gelatinase activator (MMP-14) in actinic keratosis and in in situ and invasive squamous cell carcinoma. Am J Dermatopathol 2012;34:723–728. 22534634 10.1097/DAD.0b013e31824b1ddf

[51] Park JH, Jo JH, Kim KH, Kim SJ, Lee WR, Park KK, et al Antifibrotic effect through the regulation of transcription factor using ring type-Sp1 decoy oligodeoxynucleotide in carbon tetrachloride-induced liver fibrosis. J Gene Med 2009;11:824–833. 10.1002/jgm.1355 19554625

[52] Storz P, Doppler H, Copland JA, Simpson KJ, Toker A. FOXO3a promotes tumor cell invasion through the induction of matrix metalloproteinases. Mol Cell Biol 2009;29:4906–4917. 10.1128/MCB.00077-09 19564415 PMC2738298

[53] Kim ES, Moon A. Role of transforming growth factor-β in tumor invasion and metastasis. Toxicol Res 2007;23:197–205.

[54] Piao MJ, Susara Ruwan Kumara MH, Kim KC, Kang KA, Kang HK, Lee NH, et al Diphlorethohydroxycarmalol suppresses ultraviolet B-induced matrix metalloproteinases via inhibition of JNK and ERK signaling in human keratinocytes. Biomol Ther 2015;23:557–563. 10.4062/biomolther.2015.054 PMC4624072 26535081

[55] Buters JT, Sakai B, Richter T, Pineau T, Alexander DL, Savas U, et al Cytochrome P450 CYP1B1 determines susceptibility to 7, 12-dimethylbenz[a]anthracene-induced lymphomas. Pro Natl Acad Sci U S A 1999;96:1977–1982. 10.1073/pnas.96.5.1977 PMC26722 10051580

[56] Page TJ O’Brien S, Holston K, MacWilliams PS, Jefcoate CR, Czuprynski CJ. 7,12-Dimethylbenz[a]anthracene-induced bone marrow toxicity is p53-dependent. Toxicol Sci 2003;74:85–92. 12730609 10.1093/toxsci/kfg115

[57] Buters JT, Mahadevan B, Quintanilla-Martinez L, Gonzalez FJ, Greim H, Baird WM, et al Cytochrome P450 1B1 determines susceptibility to dibenzo[a,l]pyrene-induced tumor formation. Chem Res Toxicol 2002;15:1127–1135. 12230405 10.1021/tx020017q

[58] Galvez-Peralta M, Shi Z, Chen J, Miller ML, Nebert DM. Oral benzo[a]pyrene in Cyp1a1/1b1(-/-) double-knockout mice: Microarray analysis during squamous cell carcinoma formation in preputial gland duct. Int J Cancer 2013;132:2065–2075. 10.1002/ijc.27897 23047765

[59] Tsuchiya Y, Nakajima M, Yokoi T. Critical enhancer region to which AhR/ARNT and Sp1 bind in the human CYP1B1 gene. J Biochem 2003;133:583–592. 12801909 10.1093/jb/mvg075

[60] Maillot G, Lacroix-Triki M, Pierredon S, Gratadou L, Schmidt S, Benes V, et al Widespread estrogen-dependent repression of micrornas involved in breast tumor cell growth. Cancer Res 2009;69:8332–8340. 10.1158/0008-5472.CAN-09-2206 19826037

[61] Ding L, Xu Y, Zhang W, Deng Y, Si M, Du Y, et al MiR-375 frequently downregulated in gastric cancer inhibits cell proliferation by targeting JAK2. Cell Res 2010;20:784–793. 10.1038/cr.2010.79 20548334

[62] Wang F, Li Y, Zhou J, Xu J, Peng C, Ye F, et al miR-375 is down-regulated in squamous cervical cancer and inhibits cell migration and invasion via targeting transcription factor SP1. Am J Pathol 2011;179:2580–2588. 10.1016/j.ajpath.2011.07.037 21945323 PMC3204087

[63] Liu AM, Poon RT, Luk JM. MicroRNA-375 targets Hippo-signaling effector YAP in liver cancer and inhibits tumor properties. Biochem Biophys Res Commun 2010;394:623–627. 10.1016/j.bbrc.2010.03.036 20226166

[64] Nishikawa E, Osada H, Okazaki Y, Arima C, Tomida S, Tatematsu Y, et al miR-375 is activated by ASH1 and inhibits YAP1 in a lineage-dependent manner in lung cancer. Cancer Res 2011;71:6165–6173. 10.1158/0008-5472.CAN-11-1020 21856745

[65] Li X, Lin R, Li J. Epigenetic silencing of microRNA-375 regulates PDK1 expression in esophageal cancer. Dig Dis Sci 2011;56:2849–2856. 10.1007/s10620-011-1711-1 21533613

[66] Ward A, Balwierz A, Zhang JD, Kublbeck M, Pawitan Y, Hielscher T, et al Re-expression of microRNA-375 reverses both tamoxifen resistance and accompanying EMT-like properties in breast cancer. Oncogene 2013;32:1173–1182. 10.1038/onc.2012.128 22508479

