# Supplementary material for: CYP1B1 Enhances Cell Proliferation and Metastasis through Induction of EMT and Activation of Wnt/β-Catenin Signaling via Sp1 Upregulation
Source: PLoS One. 2016 Mar 16;11(3):e0151598. doi: 10.1371/journal.pone.0151598 (PMC4794175; doi:10.1371/journal.pone.0151598)
Supplement: S1 Table — (DOCX) [file pone.0151598.s006.docx]

**Supplementary Table S1.** Primers for quantitative realtime-PCR (qPCR) analysis.

| CYP1B1  Sense  Antisense | 5'-CACTGCCAACACCTCTGTCTT-3’  5'-CAAGGAGCTCCATGGACTCT-3’ |  |
| --- | --- | --- |
| Sp1  Sense  Antisense | 5'-TGGCAGCAGTACCAATGGC-3’  5'-CCAGGTAGTCCTGTCAGAACTT-3’ |  |
| β-catenin  Sense  Antisense | 5'- ATGTCCAGCGTTTGGCTGAA -3’  5'- TGGTCCTCGTCATTTAGCAGTT -3’ |  |
| C-myc  Sense  Antisense | 5'- GGCTCCTGGCAAAAGTCA -3’  5'- AGTTGTGCTGATGTGTGGAGA -3’ |  |
| Cyclin D1  Sense  Antisense | 5'- ACCTGAGGAGCCCCAACA -3’  5'- TCTGCTCCTGGCAGGCC -3’ |  |
| E-cadherin  Sense  Antisense | 5'- AAAGGCCCATTTCCTAAAAACCT -3’  5'- TGCGTTCTCTATCCAGAGGCT -3’ |  |
| N-cadherin  Sense  Antisense | 5'- GGTGGAGGAGAAGAAGACCAG -3’  5'- GGCATCAGGCTCCACAGT -3’ |  |
| SNAI1  Sense  Antisense | 5'- CTTCCAGCAGCCCTACGAC -3’  5'- CGGTGGGGTTGAGGATCT -3’ |  |
| ZEB1  Sense  Antisense | 5'- TCCTGAGGCACCTGAAGAGG -3’  5'- CAGAGAGGTAAAGCGTTTATAGCC -3’ |  |
| ZEB2  Sense  Antisense | 5'- AAAACCATGGCGTGGGTA -3’  5'- CAATAGCCGAGGCATCAAC-3’ |  |
| TWIST1  Sense  Antisense | 5'- GGGAGTCCGCAGTCTTAC -3’  5'- CCTGTCTCGCTTTCTCTTT -3’ |  |
| α-SMA  Sense  Antisense | 5'- CAGGGCTGTTTTCCCATCCAT -3’  5'- ACGTAGCTGTCTTTTTGTCCC -3’ |  |
| vimentin  Sense  Antisense | 5'- CCCTCACCTGTGAAGTGGAT -3’  5'- TCCAGCAGCTTCCTGTAGGT -3’ |  |
| fibronectin  Sense  Antisense | 5'- CCGTGGGCAACTCTGTC -3’  5'- TGCGGCAGTTGTCACAG-3’ |  |
| integrin α5  Sense  Antisense | 5'- GCCTGTGGAGTACAAGTCCTT-3’  5'- AATTCGGGTGAAGTTATCTGTGG -3’ |  |
| α-catenin  Sense  Antisense | 5'- CTGTTGGAGCCTCTTGTTAC-3’  5'- CATTACACTCTGCCACAATTC-3’ |  |
| MMP1  Sense  Antisense | 5'- TGGACCATGCAATTGAGAAA -3’  5'- CCGATGATCTCCCCTGACAA -3’ |  |
| MMP9  Sense  Antisense | 5'- GTGCTGGGCTGCTGCTTTGCTG -3’  5'- GTCGCCCTCAAAGGTTTGGAAT -3’ |  |
| MMP13  Sense  Antisense | 5'- CCAGACTTCACGATGGCATTG-3’  5'- GGCATCTCCTCCATAATTTGGC -3’ |  |
| MMP14  Sense  Antisense | 5'- CATCTGTGACGGGAACTTTGA-3’  5'- GGCAGTGTTGATGGACGCA-3’ |  |
| GAPDH  Sense  Antisense | 5'- TCCACTGGCGTCTTCACC -3’  5'- GGCAGAGATGATGACCCTTTT -3’ |  |
